# Diagnosis of alveolar and root fractures: an *in vitro* study comparing CBCT imaging with periapical radiographs

**DOI:** 10.1590/1678-77572016-0332

**Published:** 2017

**Authors:** Solange KOBAYASHI-VELASCO, Fernanda Cristina Sales SALINEIRO, Ivan Onone GIALAIN, Marcelo Gusmão Paraiso CAVALCANTI

**Affiliations:** 1 Universidade de São Paulo, Faculdade de Odontologia, Departamento de Estomatologia, São Paulo, SP, Brasil.

**Keywords:** Diagnostic imaging, Cone-beam computed tomography, Digital dental radiography, Tooth fractures, Bone fractures

## Abstract

**Objective:**

To compare periapical radiograph (PR) and cone-beam computed tomography (CBCT) in the diagnosis of alveolar and root fractures.

**Material and Methods:**

Sixty incisor teeth (20 higid and 40 with root fracture) from dogs were inserted in 60 anterior alveolar sockets (40 higid and 20 with alveolar fracture) of 15 macerated canine maxillae. Each fractured socket had a root fractured tooth inserted in it. Afterwards, each maxilla was submitted to PR in two different vertical angulation incidences, and to CBCT imaging with a small field of view (FOV) and high-definition protocol. Images were randomized and posteriorly analyzed by two oral and maxillofacial radiologists two times, with a two-week interval between observations.

**Results:**

Sensitivity and specificity values were good for root fractures for PR and CBCT. For alveolar fractures, sensitivity ranged from 0.10 to 0.90 for PR and from 0.50 to 0.65 for CBCT. Specificity for alveolar fractures showed lower results than for root fractures for PR and CBCT. Areas under the ROC curve showed good results for both PR and CBCT for root fractures. However, results were fair for both PR and CBCT for alveolar fractures. When submitted to repeated measures ANOVA tests, there was a statistically significant difference between PR and CBCT for root fractures. Root fracture intraobserver agreement ranged from 0.90 to 0.93, and alveolar fracture intraobserver agreement ranged from 0.30 to 0.57. Interobserver agreement results were substantial for root fractures and poor/fair for alveolar fractures (0.11 for PR and 0.30 for CBCT).

**Conclusion:**

Periapical radiograph with two different vertical angulations may be considered an accurate method to detect root fractures. However, PR showed poorer results than CBCT for the diagnosis of alveolar fractures. When no fractures are diagnosed in PR and the patient describes pain symptoms, the subsequent exam of choice is CBCT.

## Introduction

Trauma on the anterior region of maxilla may commonly result in tooth and/or alveolar fracture. In children, trauma factors may be associated with accidental falls, contact sports injuries or wheeled toys such as bicycles, skateboards, scooters or roller skates. When the dental fracture happens in the crown, it is clinically diagnosed and the periapical radiograph (PR) is used to evaluate its extension and proximity to the pulp. However, in cases of root fracture, PR or cone beam computed tomography (CBCT) images may be used to confirm the fracture and observe the tooth (crown and root) and adjacent alveolar bone. While analyzing the images, it is essential to locate the root region (cervical, medium or apical third), the root fracture line direction (horizontal, oblique or vertical), the alveolar fracture location (buccal or palatine/lingual) and size of the osseous fragment to determine treatment and prognosis at the affected area^12,20,23-25,30^.

Periapical radiographs (PR) are an important diagnostic tool for general practitioners and dental specialists likewise. However, this method presents a two-dimensional image, not allowing the observation of buccal and lingual (or palatine) regions and resulting in superimposition of structures^8^.

CBCT is an imaging modality that enables a three-dimensional analysis of dental and bone structures in the oral cavity. This method permits a more accurate interpretation of dental and alveolar injuries, allowing the dentist to analyze the area of interest through multiplanar reconstructed images (axial, coronal and sagittal planes)^4^.

The purpose of this study was to compare PR with CBCT imaging for the diagnosis of alveolar and horizontal root fractures by using an *in vitro* model (macerated canine maxillae).

## Material and methods

### Preparation of samples

Sixty incisor teeth from the canine species (*Canis lupus familiaris*) were inserted in 60 anterior alveolar sockets of 15 macerated canine maxillae for this study. The sockets were previously inspected for absence of fractures and the teeth were inspected for absence of cavities, root resorption or fractures.

The Ethics Committee for the Use of Animals at our institution exempted this research from approval under the protocol number 011/2015.

One operator randomly divided each maxilla site (60 sites in total) in three groups:

Group 1: higid tooth and higid alveolar socket (20 sites);

Group 2: fractured root and higid alveolar socket (20 sites);

Group 3: fractured root and fractured alveolar socket (20 sites).

This randomization was originated through the website www.random.org (Randomness and Integrity Services Ltd, Dublin, Ireland).

**Figure 1:**
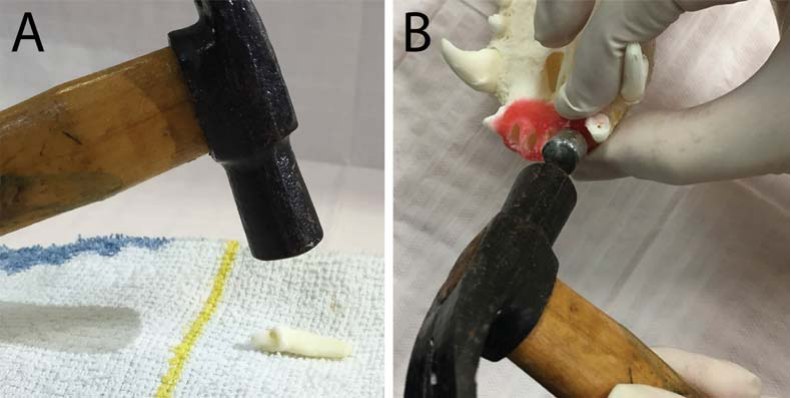
A: Root fracture produced with hammer; B: Alveolar fracture produced with metal bolt and hammer

The same operator, who was not involved in interpreting the images, induced the root fracture in 40 teeth. Each tooth was placed on a horizontal soft foundation^7,16,27^and a hammer was used to apply a perpendicular force. The fragments were glued with cyanoacrylate (Henkel, São Paulo, SP, Brazil) as previously described by Costa, et al.^7^ (2011) (Figure 1A).

For the alveolar fracture, a novel technique was developed. First, the maxillae were prepared with modelling wax (Lysanda, São Paulo, SP, Brazil) around the alveolar process. This step was important to keep bone fragments in position after the fracture (mimicking the gums) during the image acquisition. The operator inserted an individualized metal bolt (with its diameter smaller than the alveolar socket diameter) inside the toothless socket. Another person maintained the maxilla in position and held buccal and palatine cortices with the thumb and index finger. The first operator applied a force at the bolt head with a hammer. The bolt tip produced a pressure at the bone resulting in the alveolar fracture (Figure 1B). Confirmation of the fracture was made by visual inspection. The fractured tooth was then inserted in the socket.

### Image acquisition

The operator who induced the root fracture also acquired all the periapical radiographs and the CBCT images. Digital PRs were obtained by using Focus (Instrumentarium Dental, Tuusula, Finland) intraoral X-ray at 7mA, 60 kVp and 0.06 seconds of exposure time, VistaScan (Durr Dental, Bietigheim-Bissingen, Germany) intraoral phosphor storage plates (PSP) and PerioPlus (Durr Dental, Bietigheim-Bissingen, Germany) system. For each maxilla, two images were acquired: the first one parallel to the tooth long axis and the second one in a 10 degree vertical angle variation increase towards the crown (in such a way that the observers had two different images to analyze), similarly to the technique described by Celikten, et al.^5^ (2014).

CBCT images were acquired at PlanmecaProMax (Planmeca Oy, Hellsinki, Finland) high definition (HD) tooth protocol with the following parameters: 80 kV, 8 mA, field of view (FOV) 5x5.5 cm, voxel 0.15 mm and 500 frames. Condensation silicone dental putty (Optosil, Heraeus-Kulzer, Hanau, Germany) was used to position the maxilla to simulate the patient’s positioning at the tomography.

The authors opted for comparing PR with CBCT images because CBCT is a very widespread imaging modality in dentistry^6^.

### Radiographic assessment

Observation sequences for both PR and CBCT images were randomized through a website (www.random.org, Randomness and Integrity Services Ltd, Dublin, Ireland). Two blind previously calibrated and CBCT trained oral and maxillofacial radiologists used the same workstation independently to perform the analyses. In order to assess intraobserver agreement, all images were evaluated after a two-week interval.

Periapical images were observed on an iMac 27” Mac OS X (Apple, Cupertino, USA) workstation, in 15 randomized groups. Each group contained two images of the same maxilla (the first image perpendicular to the long axis and the second one on a 10 degree vertical angle variation towards the crown). The observers had to identify the root fracture (yes/no), and the presence of alveolar fracture (yes/no).

CBCT images were imported into OsiriX 3.8.1 (Pixmeo, Geneva, Switzerland; http://www.osirix-viewer.com/), an open-source DICOM viewer for MacOS. Observers could use all software features to identify root fracture (yes/no) and presence of alveolar fracture (yes/no). The observers interpreted the volume data using multiplanar reconstructed images (axial, coronal and sagittal) simultaneously.

### Data analysis

The presence of a tooth fracture line was classified as 0 (absence of fracture) and 1 (presence of fracture) and compared with the gold standard (visual inspection). All data were tabulated and inserted into MedCalc (MedCalc Software, Ostend, Belgium) software. Sensitivity, specificity and area under the receiver operating characteristic (ROC) curve were independently calculated for each observation performed by observers 1 and 2. Subsequently, areas under the ROC curve were compared by using repeated measures ANOVA test. The same rationale was used for the alveolar fractures.

Statistical analyses were performed using kappa tests. Kappa coefficient was calculated to assess the degree of intra- and interobserver agreement, and scored as poor agreement (0-0.19), fair agreement (0.20-0.39), moderate agreement (0.40-0.59), substantial agreement (0.60-0.79) and almost perfect agreement (0.80-1.00)^19^. Kappa data were analyzed using the website www.lee.dante.br (Epidemiology and Statistics Laboratory, Dante Pazzanese Institute of Cardiology, São Paulo, SP, Brazil).

## Results

This study included 60 sites in 15 dogs anterior maxillae. Twenty sites had neither alveolar nor root fracture, 20 comprised only root fracture and 20 enclosed both root and alveolar fractures.

Table 1 [Table t2] shows the results for sensitivity, specificity and area under the ROC curve for both periapical radiographs and CBCT images, for root fractures and alveolar fractures independently. Sensitivity values were good for root fractures for both PR (ranging from 0.78 to 0.85) and CBCT (ranging from 0.83 to 0.93). For alveolar fractures, values ranged from 0.10 to 0.90 for PR and from 0.50 to 0.65 for CBCT. Specificity numbers followed the same trend for root fractures, ranging from 0.85 to 0.95 for PR and from 0.95 to 1.00 for CBCT. When observing alveolar fractures, variations were similar for both PR (ranging from 0.50 to 0.55) and CBCT (ranging from 0.55 to 0.78).

**Table 1:** Sensitivity (Se), Specificity (Sp) and Area under the ROC curve (AUR) values for root and alveolar fractures, for both observers

		Root Fracture						Alveolar fracture					
		First Observation			Second Observation			First Observation			Second Observation		
		Se	Sp	AUR	Se	Sp	AUR	Se	Sp	AUR	Se	Sp	AUR
Observer 1	Periapical	0.83	0.95	0.89	0.85	0.85	0.85	0.1	0.9	0.5	0.85	0.18	0.51
	CBCT	0.9	0.95	0.93	0.93	1.00	0.96	0.5	0.73	0.61	0.65	0.9	0.78
Observer 2	Periapical	0.78	0.9	0.84	0.8	0.95	0.88	0.9	0.13	0.51	0.35	0.75	0.55
	CBCT	0.83	0.95	0.89	0.85	0.95	0.9	0.5	0.6	0.55	0.5	0.8	0.65

**Table 2 t2:** Kappa test values. Inter- and intraobserver concordances on diagnosis of root and alveolar fractures

Image	Observer 1 X Observer 1		Observer 2 X Observer 2		Observer 1 X Observer 2	
	Root Fracture	Alveolar Fracture	Root Fracture	Alveolar Fracture	Root Fracture	Alveolar Fracture
Periapical	0.93	0.3	0.9	0.57	0.75	0.11
CBCT	0.9	0.45	0.93	0.54	0.74	0.3

Areas under the ROC curve (AUR) showed good results for both PR (ranging from 0.84 to 0.89) and CBCT (ranging from 0.89 to 0.96) when analyzing root fractures. For alveolar fractures, however, results were fair, ranging from 0.50 to 0.55 for PR and from 0.55 to 0.78 for CBCT. AUR values were submitted to repeated measures ANOVA tests, in order to statistically determine significant differences between PR and CBCT, for root and alveolar fractures independently. The ANOVA tests resulted in statistically significant differences between PR and CBCT for root fractures only.

Table 1 shows kappa values for both root and alveolar fractures. Root fracture intraobserver agreement ranged from 0.90 to 0.93 (similar results were obtained for PR and CBCT), and alveolar fracture intraobserver agreement ranged from 0.30 to 0.57. Interobserver agreement results were substantial for root fractures (0.74-0.75) and poor/fair for alveolar fractures (0.11 for PR and 0.30 for CBCT).

## Discussion

It is essential to select the most adequate radiographic exams for the patient when facing dental and maxillofacial injuries^11^. PR consists of a 2D image of a 3D object resulting in superimposition of images, hence not allowing a thorough observation of buccal and palatine/lingual regions. Also, the diagnosis of root fracture is more difficult when the x-ray beam is not parallel to the fracture line^26^. A three-dimensional imaging method allows a more thorough visualization because it eliminates superimposition of structures^4^.

**Figure 2:**
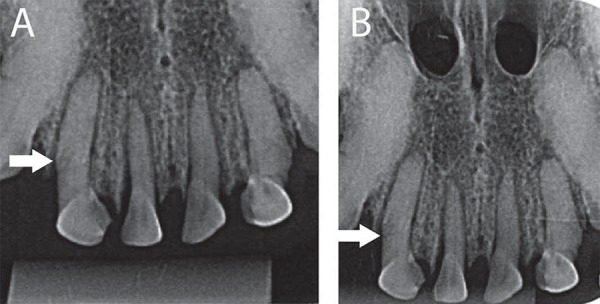
A: Periapical radiograph of the right lateral incisor, showing root fracture (filled arrow). Orthogonal angulation; B: Periapical radiograph of the right lateral incisor, demonstrating root fracture (filled arrow). 10º vertical angulation from orthogonal

**Figure 3:**
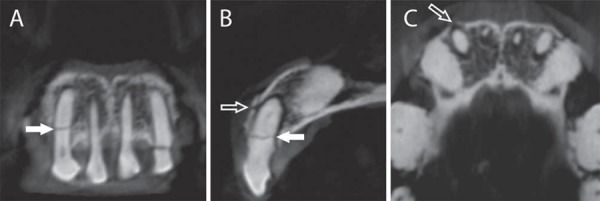
A: CBCT coronal image of the right lateral incisor, showing root fracture (filled arrow); B: CBCT sagittal image of the right lateral incisor, depicting root fracture (filled arrow) and alveolar fracture (outlined arrow); C: CBCT axial image of the right lateral incisor, showing alveolar fracture (outlined arrow)

In PR, the observers were able to visualize a root fracture but did not detect an alveolar fracture (Figures 2A and 2B). However, both root and alveolar fractures were observed in CBCT images. The root fracture was demonstrated in coronal and sagittal planes, and the alveolar fracture was shown in sagittal and axial planes (Figures 3A, 3B, 3C).

Canine species (*Canis lupus familiaris*) has been used in studies in dentistry^13,17,21^ and its anterior maxilla and dental anatomy resemble the human one.

Several studies, both *in vitro* and *in vivo* ^2,11,16-18^, affirmed that CBCT is a more accurate diagnostic method when compared with periapical radiographs for diagnosis of root fracture. Bechara, et al.^2^ (2013) compared photostimulated phosphor plate (PSP) images with CBCT for the detection of root fracture on endodontically treated teeth. The authors concluded that small FOV images had a higher accuracy and sensitivity when compared with PSP images. In our study, for the CBCT exam, we used a small FOV and a small voxel [based on the findings of (2016), Salineiro, et al.^26^(2015) and Bechara, et al.^2^ (2013)], and also the largest number of frames that the machine allowed [based on the findings of Costa, et al.^9^ (2014)] and obtained slightly superior sensitivity and accuracy levels for CBCT, when compared with periapical radiographs for the detection of root fracture. We believe that these values are explained by the fact that two periapical images were concomitantly analyzed, which allowed the observers to better identify root fractures by providing two different x-ray incidences. This fact enlightens the value of analyzing periapical radiographs in two different angulations on detecting root fractures.

Ilguy, et al.^18^ (2009) reported a case where a panoramic radiograph and a posteroanterior radiograph were used as first diagnostic methods for a trauma patient. These images showed bilateral condyle and a left mandibular incisor region fractures and no dental fractures. After the patient was submitted to a CBCT exam, alveolar fracture lines and a root fracture were also diagnosed. This study showed the importance of CBCT in identifying smaller size fractures, corroborating our findings and results.

Segmental displacements of alveolar bone can be clinically observed^1,29^and additional imaging exams are needed to provide further information such as fracture location, extension and relationship with important anatomical structures. A few case reports described the diagnosis of alveolar fracture by using radiographs^3,10^ while some other case reports affirmed that CBCT is an effective diagnostic method for minor alveolar fractures^11,15,18^. Our study showed lower AUR numbers in the detection of alveolar fractures, when compared with root fractures, by CBCT. We also noticed a slight increase in the second observation for alveolar fracture. These variations may be explained by the vast amount of large diameter haversian canals in canine maxillae when compared with human maxillae, and also by the methodology used to perform alveolar fractures that, in some cases, resulted in incomplete fractures. Both occurrences may have produced misdiagnosed cases of alveolar fractures and poor/fair interobserver agreement.

Another point to be observed is the ALARA (as low as reasonably achievable) principle^14^, by which the professional will adopt CBCT only in cases in which the periapical radiograph will not suffice to reach a correct diagnosis. According to Loubele, et al.^22^(2009), radiation dose needs to be kept as low as possible, however allowing a good quality image for the diagnosis. In our results, we observed a propensity to slightly higher results with CBCT, when compared with periapical radiographs. We believe that CBCT exam is necessary in cases in which the periapical radiograph does not show a fracture line but the patient shows symptoms that could be associated with root and/or alveolar fracture. Hence, the clinician has to balance its harmfulness with its efficiency to prescribe this exam^6^.

According to Yamamoto, et al.^28^ more studies are needed to analyze the long-term clinical implications of alveolar fracture such as location, severity, and stage of child development when the fracture occurred. Thus, a correct imaging diagnosis is essential for a better prognosis and treatment outcome.

## Conclusions

Periapical radiographs with two different vertical angulations may be considered an accurate method to detect root fractures. However, PR showed lower results for the diagnosis of alveolar fractures when compared with CBCT imaging. When no fractures are diagnosed in PR and the patient describes pain symptoms, the subsequent exam of choice is CBCT.
